# Age- and sex-specific incidence rates and future projections for hip fractures in The Gambia, West Africa, and comparison across four countries in Africa

**DOI:** 10.1093/jbmr/zjaf126

**Published:** 2026-07-24

**Authors:** Hannah Wilson, Kebba Marenah, Anya Burton, Momodou Jallow, Lucy Gates, Awa Touray, Samuel Hawley, Simon M. Graham, James Masters, Matthew Costa, Bintou Trawally, Kate A. Ward, Celia L. Gregson

**Affiliations:** 1 Musculoskeletal Research Unit, University of Bristol, Bristol BS10 5NB, United Kingdom; 2 Department of Orthopaedics and Trauma, Edward Francis Small Teaching Hospital, Banjul, The Gambia; 3 MRC Unit The Gambia at London School of Hygiene and Tropical Medicine, The Gambia; 4 MRC Lifecourse Epidemiology Centre, School of Human Development and Health, University of Southampton, Southampton, SO16 6YD, United Kingdom; 5 Oxford Trauma and Emergency Care, Nuffield Department of Orthopaedics, Rheumatology and Musculoskeletal Science, University of Oxford, Oxford OX3 7LD, United Kingdom

**Keywords:** hip fracture incidence, The Gambia, West Africa, hip fracture projections, fragility fractures

## Abstract

Longevity in African populations is increasing, where deprivation and malnutrition are common; hence, fragility fracture incidence is expected to increase. Healthcare systems must adapt to provide for these aging populations; however, currently fragility fracture incidence has yet to be determined in any West African setting. This study aimed to determine age- and sex-specific hip fracture incidence rates in adults in The Gambia, compare these with rates from other African countries, and estimate future national hip fracture projections. All hip fracture cases in adults aged ≥40 yr, presenting to a hospital or traditional bone setter (TBS) in the study area over 2-yr, were identified. Age- and sex-specific hip fracture incidence per 100 000 person-years were estimated using the 2024 Gambian Population census. Incidence rate estimates were compared between The Gambia, Zimbabwe, South Africa, and Botswana. In The Gambia, future hip fracture numbers were estimated through to 2054 using United Nations population projections. Over 2-yr, 226 hip fracture patients, mean (SD) age 71.2 (12.5) yr, 64.6% female, presented to hospital (184 [81.4%]) or TBS (42 [18.6%]). Most presented with a fragility fracture (205 [90.7%]). High-impact trauma (eg, traffic accidents) was more common in younger men. Delays in presentation were common (68 [30.1%]). Incidence rates for adults ≥40 yr in The Gambia were 28.1 and 51.7 per 100 000 person years for men and women, respectively, increasing with age. In those age ≥80 yr, incidence rates plateaued in men. The number of hip fractures is estimated to increase from 166 in 2024 to 621 by 2054. Age-specific hip fracture incidence rates were broadly comparable between The Gambia, Zimbabwe, Botswana, and Black South Africans. In summary, fragility fractures in Gambian adults were common, indicative of age-associated osteoporosis. Hip fracture cases will almost quadruple over coming decades; therefore, health service capacity must expand to manage this rising demand.

## Introduction

Longevity in African populations is increasing.^1^ Across the continent, age-related comorbidities are more commonly seen; this includes musculoskeletal disease, which confers an increased risk of falls and fragility fractures.^2–4^ Available data from South Africa suggest that population aging will lead to a doubling in the number of fragility fractures of the hip in the Black South African population in the next 30 yr.^2^ Although current South African hip fracture incidence rates are similar to those in other middle-income countries, rates vary considerably by ethnicity, with lower rates in Black South Africans.^5,6^ Emerging data from other predominately Black African populations in neighboring Zimbabwe and Botswana have found similar incidence rates to Black South Africans.^7,8^

However, within the sub-Saharan African region, there are currently no comparable incidence data available outside of Southern Africa.

West Africa is a highly populous region of approximately 460 million people,^9^ where 40% of the sub-Saharan African population reside.^10^ This setting is characterized by a range of factors that potentially increase fragility fracture risk; socioe-conomic deprivation and malnutrition are common,^11,12^ as are rapid urbanization and road traffic trauma.^13,14^ The Gambia is one of the lowest-income countries in the world, and its demographics and tribes are broadly representative of the West African region. In this region, fracture care is often first sought from traditional bone setters (TBS); many of whom learnt their skills from older relatives rather than through formal training. Integration of TBS practices with allopathic healthcare systems can be complex.^15^

Fragility fractures lead to multiple adverse health outcomes and high healthcare costs, even within well-resourced health and social care environments.^16,17^ In low-resource settings the minimal evidence currently available suggests that fracture outcomes may be worse and costs significantly higher relative to better resourced settings.^18^ In South Africa, the average direct health cost attributable to hip fracture has been estimated as US$6395; 6-fold greater than the mean per capita health expenditure for the country.^19^

Historically, health care systems in West Africa have been designed to provide for maternal and child health and infectious diseases. These same systems now need to evolve to provide for an aging, often multimorbid, population. To plan healthcare delivery, robust epidemiological data are needed to quantify current fragility fracture incidence and estimate future burdens on the healthcare economy. Furthermore, such data can be used to calibrate clinical risk assessments tools, to help guide deployment of interventions to reduce fracture risk. This study aimed to determine age- and sex-specific hip fracture incidence rates in adults aged 40 yr and older in The Gambia, to compare these rates with those from other sub-Saharan African countries (where available), and to estimate the future number of hip fractures expected in The Gambia, in the knowledge that findings will be informative to the wider West African region.

## Materials and methods

### Study design and population

A prospective incidence study was conducted of hip fractures in The Gambia (total population 2.42 million), as part of a wider program of research into fractures in Africa.^20^ Study areas included the urban/peri-urban areas of Banjul, Kanifing, and Brikama (population 1 556 937 in 2024), and the rural region of West Kiang, which is located within Mansakonko (population 90 624 in 2024).^21^ These areas were selected to be representative of the diversity of urban and rural populations in The Gambia, as well as being accessible from our coordinating MRC Gambia centers by 4-wheel drive. In these areas, hip fracture cases could present and be diagnosed at one of 16 facilities (5 public hospitals, 9 private hospitals, and 2 private X-ray imaging centers), or to any TBS practicing in the study areas. Fifteen of the 16 facilities agreed to permit on-site data collection (these included all the hospitals in the country that can surgically manage a hip fracture). The facility that did not participate was a public hospital. In the study, areas 33 of 42 TBS identified agreed to participate.

A community-based network was established, and the 16 facilities were sensitized to hip fracture identification, in discussion with facility managers, and healthcare staff. Posters were displayed, village health workers were sensitized to the study, and WhatsApp groups were established to aid timely identification of cases. Facilities and village health workers were visited or contacted at least weekly for 2 yr to identify all new potential hip fracture presentations. The 33 TBS were also sensitized to the study, with WhatsApp groups used for the TBS practitioners to contact the study team when they had a suspected hip fracture, plus the study team called weekly to confirm if anyone with hip fracture-like symptoms had presented to the TBS, including specifically “hip dislocations,” as fractures are commonly called dislocations by TBS (identified from pre-study community engagement and involvement work with TBS). Excluding tourists, any resident adult age ≥40 yr, presenting to one of the hospitals or to the TBS in the study area were eligible to be included in the study. The age of ≥40 yr corresponds to the age-range of the FRAX fracture risk assessment tool, which can be calibrated with such data.

### Data collection

Data were collected for all incident hip fractures, following an injury within the last 12 mo and presenting for the first time to one of the 16 facilities or 33 TBS between first July 2022 and first July 2024. Data were collected by trained researchers and entered directly into pre-programmed Research Electronic Data Capture (REDCap) questionnaires (hosted by the University of Bristol) with inbuilt data validation checks, working offline on Samsung Galaxy tablets. REDCap is a secure, webbased software platform designed to support data capture for research studies.^22,23^ Data included age, sex, region of residence, presentation date, delayed presentation (defined as more than 2 wk after injury), hip fracture classification (based on radiographic diagnosis), and the trauma mechanism (high energy trauma, eg, road traffic accident, or low energy trauma eg, fall from a standing height or less). Names were also collected, and stored securely locally with the patient ID. Where names on more than one record appeared similar, age, sex, region of residence, and approximate timing of injury were compared to identify duplicate records. In case of duplication only one record was kept.

### Hip fracture classification

Radiographs were used wherever possible to diagnose a hip fracture. Radiographs were reviewed independently by two orthopedic surgeons and classified as intracapsular (International Classification of Diseases, 10th Revision [ICD-10] code S72.0), pertrochanteric (ICD-10 code S72.1) or subtrochanteric (ICD-10 code S72.2); in cases of disagreement, a third senior orthopedic surgeon (M.C.) reviewed the case and consensus was reached. Where a radiograph was taken but not made available to the team, hip fracture classification was taken from the orthopedic surgical notes. In cases where there was no radiograph (eg, when treated solely by TBS, X-ray equipment faults, interrupted electricity supply, or unaffordability of radiographs) case history was used by an orthopedic surgeon to determine a clinical diagnosis of a hip fracture. Hip fractures were defined clinically following: (1) a fall where the patient landed on their buttocks or side, after which (2) they quickly experienced severe pain in the groin or hip with or without radiation down to the knee, and (3) a shortened and externally rotated leg was evident on examination.

### Statistical analysis

#### Characteristics of hip fracture patients

Characteristics of the hip fracture population were described using numbers with percentages and means with standard deviations. Chi-squared tests were used to determine associations between patient characteristics and (1) delayed presentation and (2) mechanism of injury.

#### Hip fracture incidence

The number of hip fracture cases were counted over 2 yr and the mean per year calculated; including 2 yr of data collection increased the precision of incidence estimates. The contemporaneous Gambian 2024 National Population and Housing Census provided the total population of The Gambia, stratified by sex and 5-yr age band.^21^ The Census also provided the total population of Banjul, Kanifing, and Brikama and the total population of Mansakonko which includes the West Kiang region, but not stratified by age and sex. We therefore applied the sex and age-band proportions from the total population of The Gambia to the total population of the study areas Banjul, Kanifing, Brikama, and Mansakonko.

Age- and sex-specific hip fracture incidence rates per 100 000 persons per year for the study area were calculated as the number of fractures divided by the at-risk population (adults in each age/sex strata living in the study area in 2024). Overall incidence per 100 000 adults age ≥40 yr in the study area was calculated as the total number of fractures in 1 yr divided by the estimated at-risk population (ie, all adults aged ≥40 yr living in the study area in 2024). Overall incidence in those over ≥50 yr and ≥65 yr were calculated in a similar way. Age- and sex-specific incidence rates were used to determine the relative risk ratio with 95% CI for each age band, using the 40-44 yr age band as the reference group. National hip fracture age standardized incidence rates per 100 000 persons aged ≥40, ≥50 and ≥65 per year in 2024 were calculated by applying the observed study area age- and sex-specific incidence rates to the age and sex structure of those age ≥40 yr in the whole of The Gambia in 2024. As the sex and age-band distribution in the study population had been estimated from that in the total population in the census, the incidence rates are identical. A sensitivity analysis was performed that excluded high energy trauma hip fractures to determine whether fragility fracture incidence rates were driven by high energy trauma.

#### Hip fracture projections

Yearly population projections for The Gambia, for adults age ≥40 yr, by 5-yr age band and sex, were obtained from the United Nations (UN) population prospects using the medium fertility estimates for 2024 to 2054.^24^ We have previously found that the UN population projections underestimated older age groups in South Africa and Zimbabwe compared to current census data.^2,8^ In a comparison of the UN predictions of The Gambian 2024 population to the actual 2024 population provided by the national Gambian census, the UN population estimates were higher than the Census data for age categories 40-69 and 75-79, but lower for age categories 70-74 and ≥80 yr. We therefore applied a correction factor, as we have done previously,^2,8^ to all age and sex categories from the UN population predictions from 2025 to 2054, using the proportional difference between the 2024 UN estimates and the 2024 Census data.

Hip fracture incidence rates per 100 000 persons, projected for The Gambia up to 2054, were then estimated, assuming current incidence rates remain stable. This was calculated by applying the observed study area age- and sex-specific incidence rates to the projected age and sex structure in The Gambia for each future year up to 2054. The predicted numbers of hip fractures per year were calculated by multiplying the study area age- and sex-specific incidence rates by the 5-yr age and sex band population projections and dividing by 100 000, for each future year through to 2054.

#### Cross-country comparisons using available hip fracture incidence estimates in Southern Africa

We compared the age-specific incidence rates in The Gambia, to comparable data available from prospective studies in South Africa and Zimbabwe and a retrospective study from Botswana.^6–8^ In The Gambia, 99.8% of the population is Black African,^21^ as are 99.6% of the population in Zimbabwe and 94.7% of the reported hip fracture cases in Botswana.^7,25^ We used the incidence for Black South Africans rather than the whole country to make the results more comparable.^2,6^ Statistical analyses and graphs were created using R version 4.3.3 and R Studio Version 2024.04.1+748.^26^

### Ethical and governance approvals

Ethical and governance approvals were obtained from The Gambia Government/MRC Unit The Gambia@LSHTM Scientific Coordinating Committee and Ethics committee (ref: 22975) and by The Gambian Ministry of Health. These permissions allowed collection of a minimum dataset on all hip fracture cases, from medical records and communication with staff, without the need for individual-level patient consent, to avoid selection bias.

### Community engagement and involvement

Hip fracture patients report severe pain after their injury and suffer high levels of anxiety and distress. Therefore, we did not consider it appropriate to ask for their input into this observational study; instead, we engaged multiple community-based and hospital-based healthcare personnel, village elders, and TBS to fully understand the pathway of patient care to ensure in the design of our study we maximized all chances of identifying every hip fracture in our study areas.

## Results

### Characteristics of patients with hip fracture

Over the 2-yr period, a total of 226 patients presented with a hip fracture to a facility or TBS in our study areas; most (64.2%) presented to the tertiary public hospital with the remaining presenting to a TBS or private facility, 18.6% and 17.3%, respectively (Table 1). The mean (SD) age of patients was 71.2 (12.5) yr, 64.6% were women. The study orthopedic team were able to access 190 (84.0%) radiographs to verify the ICD-10 classification; additionally, one (0.4%) patient did not have a radiograph available for the team, but classification could be inferred from surgery type. Intertrochanteric fractures were the most common (50.0%), and intracapsular second-most common (25.2%). For the 15.5% of patients who did not have a radiograph available, their diagnosis was clinical, and fracture type could not be classified; most were cases who presented to TBS (33 of 35 [94.3%]).

Delayed presentations were common with 30.1% of patients presenting more than 2 wk after their injury. Men and women were equally likely to be delayed in presentation (27 of 76 [35.5%] vs 41 of 142 [28.9%], *p* = .39), respectively. Mechanism of injury was not associated with delay in presentation (8 of 21 high-energy [38.1%] vs 60 of 197 low-energy [30.5%] injuries, *p* = .64). Older people (age ≥ 75 yr) were equally likely to be delayed in presentation as those younger (28 of 89 [31.5%] age ≥ 75 yr, vs 40 of 129 [31.0%] age 40-74 yr, *p* = 1.0). Overall, 90.7% of hip fractures were low-energy fragility fractures. Men were more likely to experience a high-energy fracture than women (18 of 80 men [22.5%] vs 3 of 146 women [2.1%], *p* < .001). A higher proportion of hip fractures were attributable to a high-energy mechanism in those who were age 40-49 yr, compared to those age ≥50 yr (9 of 15 [60.0%] 40-49 yr vs 12 of 211 [5.7%] age ≥ 50 yr, *p* < .001).

### Hip fracture incidence rates

**Figure 1:**
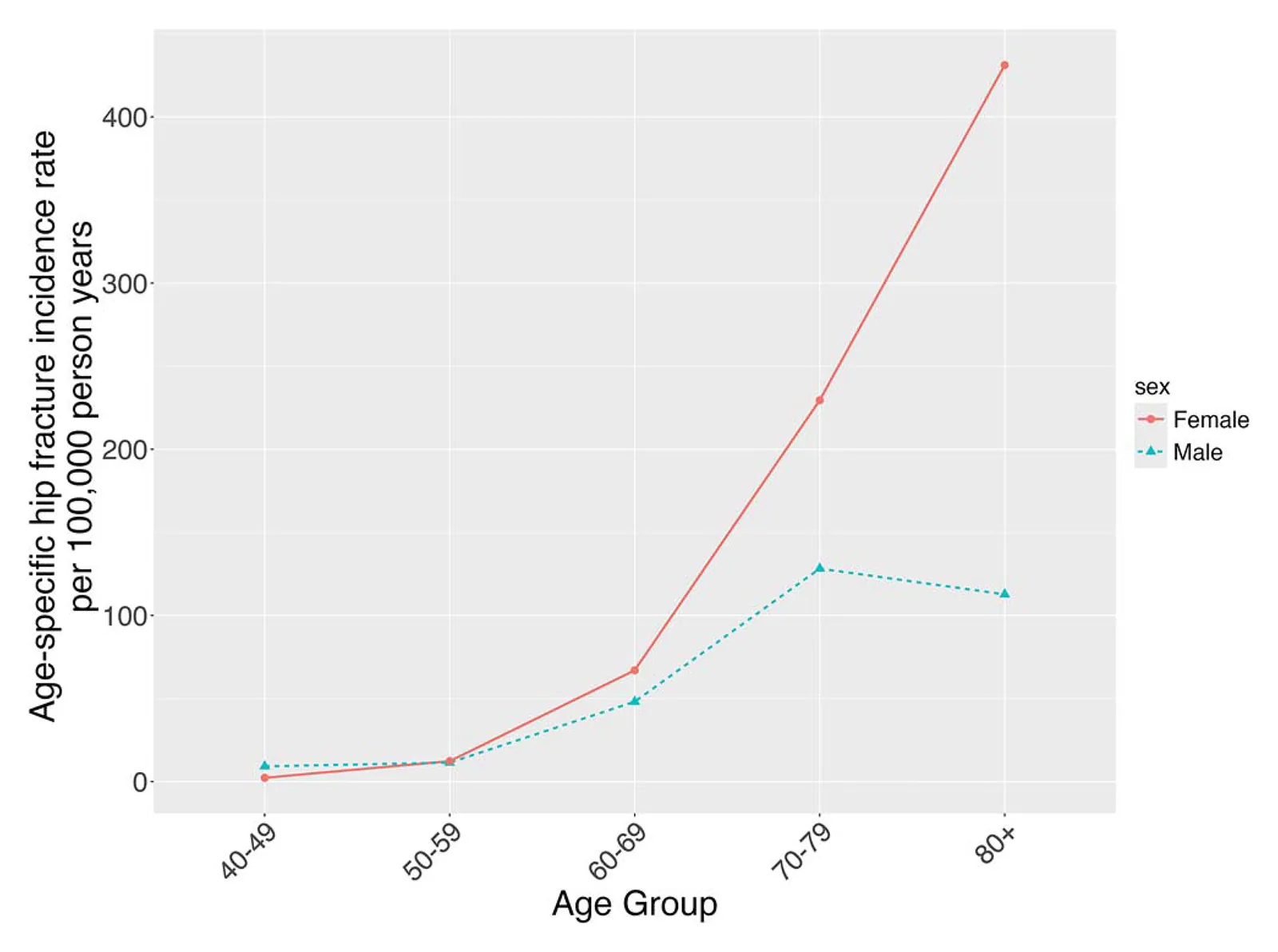
Sex- and age-specific hip fracture incidence rates per 100 000 person years in adults age ≥40 yr in The Gambia in 2024. Rates shown by 10-yr age group.

The population of adults ≥40 yr in the study areas (Banjul, Kanifing, Brikama, and Mansakonko, inclusive of West Kiang, in The Gambia) in 2024 was 283 482, representing 68% of the total population of adults ≥40 yr in The Gambia (*n* = 416 857). The hip fracture incidence rates for men and women ≥40 yr in The Gambia were 28.1 and 51.7 per 100 000 person years, respectively (Table 2). In men and women age ≥50 yr an increase in hip fracture incidence with age was evident (Figure 1, Table 2). In those 40-49 yr, hip fracture incidence was slightly higher in men than women; sensitivity analyses excluding high-energy trauma fractures confirmed this was driven by higher rates of high-energy trauma in men at this age (Figure S1). In men age ≥80 yr, incidence rates plateaued, compared to those in the younger groups.

### Projected hip fracture incidence

**Figure 2:**
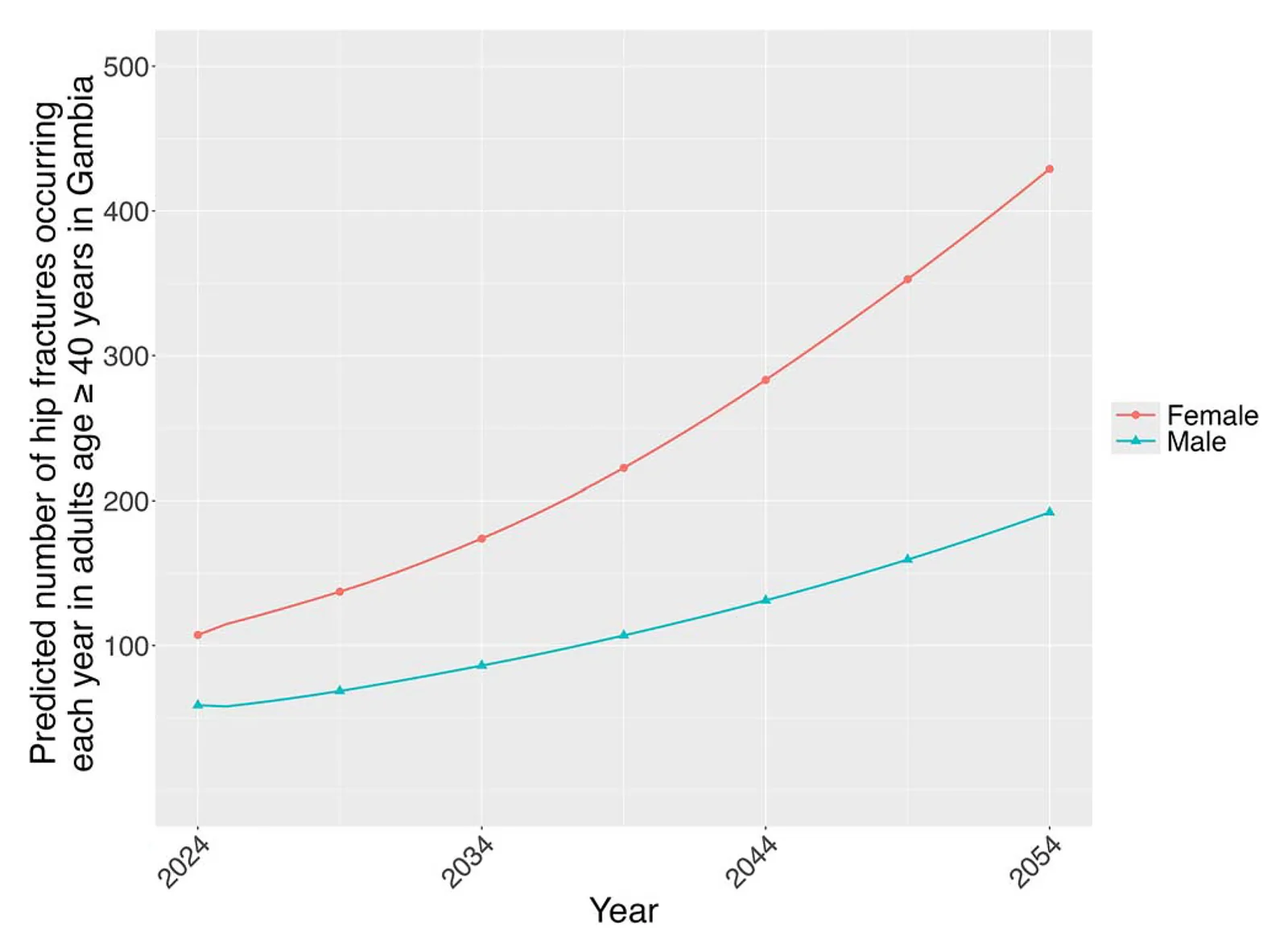
Predicted total number of hip fractures occurring in men and women each year from 2024 to 2054 in The Gambia.

The UN population predictions, with the age and sex correction factor applied, showed that adults age 40-54 yr will likely experience the greatest population growth (257 537 in 2024 growing by 165% to 682 174 in 2054) and the female population age ≥ 75 yr will experience more growth than their male counterparts (an increase of 47 326 women and 32 560 in men) (Figure S2). Across The Gambia, the absolute number of hip fractures is expected to nearly quadruple, from 166 in 2024 to 621 in 2054 (Figure 2). This increase in hip fracture numbers will be greater in women than men (in women an increase of 322 [107 in 2024 to 429 in 2054]; in men an increase of 133 [59 in 2024 to 192 in 2054]).

### Between country comparison of hip fracture incidence rates

**Figure 3:**
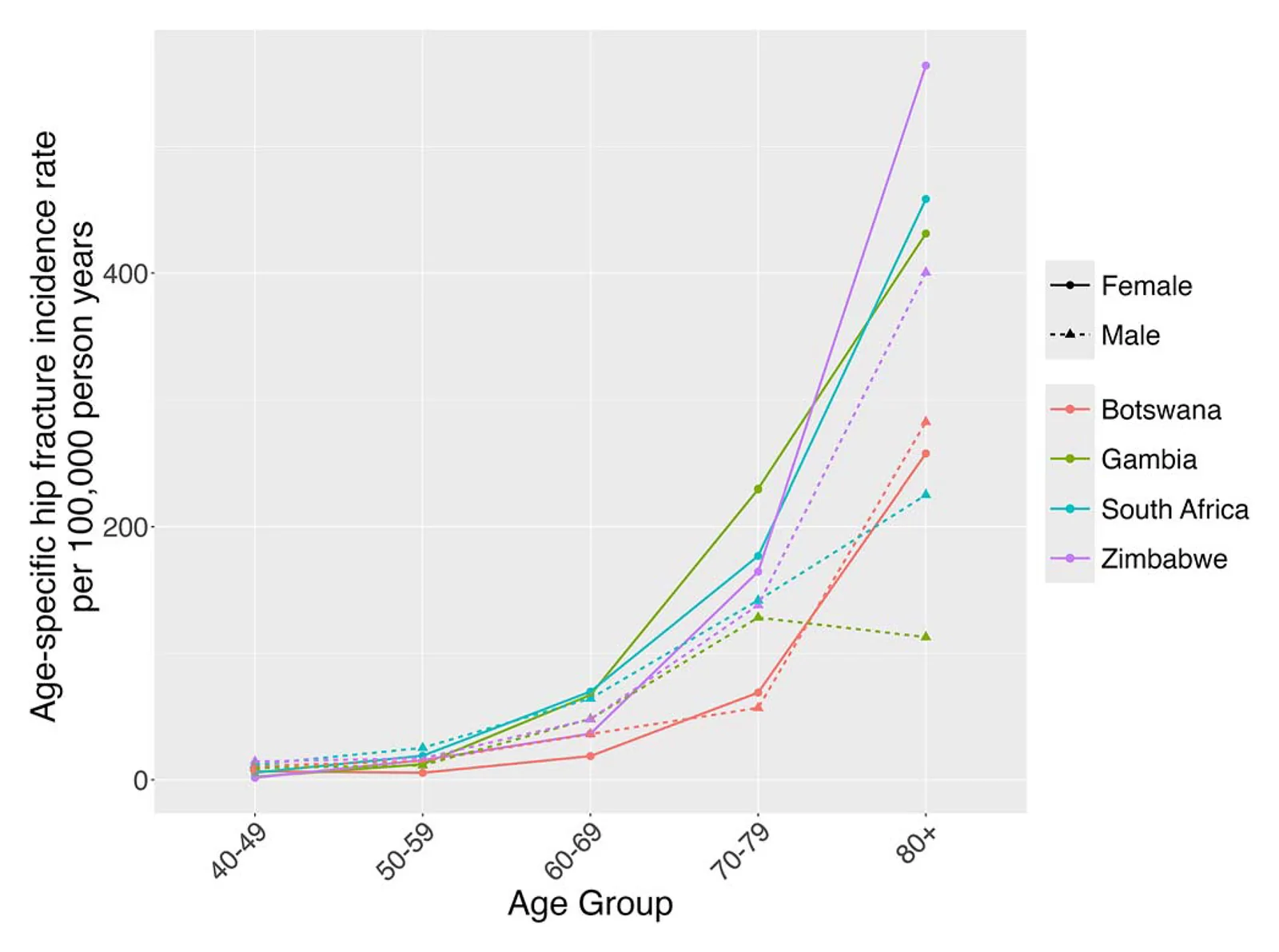
Sex- and age-specific incidence rates for hip fractures in adults age ≥40 yr in The Gambia, Zimbabwe, South Africa, and Botswana.

In those aged 40-69 yr, age-specific hip fracture incidence rates were found to be broadly comparable between The Gambia, Zimbabwe, Botswana, and the Black South African population. In those age 70 yr and older, rates were similar in Gambian, Zimbabwean, and Black South African women, and in Gambian and Black South African men (Figure 3). All countries see higher incidence in men than women in those aged 40-49 yr, (9.2 vs 2.3 per 100 000/yr in The Gambia, 14.4 vs 1.6 per 100 000/yr in Zimbabwe; 11.9 vs 5.6 per 100 000/yr in Black South Africans and 10.8 vs 6.7 per 100 000/yr in Botswana, respectively). In those age ≥ 70 yr, incidence is higher in women compared to men in The Gambia, Zimbabwe, and South Africa; in Botswana similar incidence rates were seen between men and women. The highest incidence rates were seen in Zimbabwean women age ≥80 yr.^8^

## Discussion

This is the first prospective study to determine hip fracture incidence rates in The Gambia, as well as the first in West Africa. In both men and women, most hip fractures were fragility fractures consistent with osteoporosis, and thus a clear association was seen with age. At ages 40-49 yr, the higher fracture rate seen in men, compared to women, was largely driven by high-energy fractures, such as road traffic injuries. Between the ages of 50 and 80 yr, hip fracture incidence increased to a greater extent in women than in men. Future projections estimate that hip fracture numbers in The Gambia will nearly quadruple in both men and women by 2054, putting substantial additional strain on already challenged healthcare systems that are not currently resourced to deal with this increasing demand.^27^

The hip fracture incidence rate in those age 80 yr and older unexpectedly appeared to plateau in men. We would have expected to see patterns similar to the other African countries, and countries elsewhere in the world, where the oldest in the population have the highest hip fracture incidence rates. This may reflect older men not presenting to hospitals or TBS with their hip fracture, either because they die before seeking treatment, or, for the few people over 80 yr of age living in the rural areas, it is too far, too painful, and too costly to travel to seek help.

Nearly a third (30.1%) of patients took over 2 wk to seek treatment, a significant contrast with high-income settings where surgery is recommended within 48-h of injury to improve patient outcomes.^28–30^ Delays were not associated with sex or fracture mechanism. Lack of transport and financial resource could contribute to delays in seeking treatment. Furthermore, the pathways of care in The Gambia are fluid between traditional and allopathic care; patients or their family may have consulted a TBS (outside of our study) before attending hospital, potentially delaying their presentation. Additionally, the lack of awareness and understanding of the seriousness of a hip fracture may delay presentation and onward referral by TBS. We attempted to account for this by recruiting TBS to the study and asking them to inform us of any hip fracture/injury patients who presented to them.

However, while every effort was made to recruit all TBS in the study area, a few TBS (9/42) declined to participate and some patients may have sought care from TBS in areas not included in our study sites, or in nearby countries such as Senegal.

Most hip fracture cases were fragility fractures indicative of osteoporosis.^31^ There are many barriers to clinically diagnosing osteoporosis in African countries; including insufficient clinical awareness and training (there are currently no rheumatologists or geriatricians in The Gambia), as well as lack of DXA scanning to identify those with low BMD.^4,32^ Using fracture risk assessment tools that can accurately predict fracture risk based on clinical risk factors alone (and optionally include measures of BMD if rarely they are available) will be important to target interventions to reduce future fracture burdens. The incidence data presented here will allow for the calibration of a Gambian FRAX tool.^33^ However, there are currently no anti-osteoporosis medications on the essential medicines list recommended by the WHO^34^ or the Gambian essential medicines list,^35^ and hence these are not available in The Gambia. The high number of fragility fractures found in this study highlight the need to update these global and national recommendations.

There are several strengths to our study, as it is the first prospective study of hip fracture incidence in West Africa. The inclusion of hip fracture cases presenting to both private and public hospitals (including all facilities that can treat a hip fracture), as well as those who presented to TBS increased our ability to capture all hip fracture cases, maximizing the study’s generalizability to the country as a whole, with our sampling area (Banjul, Kanifing, Brikama, and West Kiang) covering two-thirds of the total Gambian population, with urban, peri-urban, and rural regions included. Most hip fractures were confirmed radiographically, despite challenges accessing basic radiographic services in the country. Helpfully, the 2024 Gambian Population and Housing census was conducted during our study period (July 2022-July 2024),^21^ providing contemporaneous population denominators.

The main limitation of the study was that although hospitals and TBS within the study area were sensitized to the study and sustained active case finding was employed, some hip fracture cases may still have been missed, and thus incidence underestimated, particularly older rural residing persons, who never presented to a healthcare facility. For example, patients treated by TBS were not always referred onwards for radiographs or hospital care and, despite the study team’s efforts, may have been missed. It is unlikely cases were included more than once as records were checked for duplication. Second, some TBS used the terms hip fracture and hip dislocation interchangeably, thus fractures may have been under-reported. We attempted to account for this by asking TBS to refer all hip fractures and dislocations. Although most hip fractures were confirmed radiographically, lack of radiographs for TBS patients (either because they did not receive one or because they took it to their TBS before one of the orthopedic team was able to review) means that 14% of hip fracture cases lacked a radiographically confirmed hip fracture. The fluidity of patient care in The Gambia means that some patients could seek help from a TBS, a private hospital, and a public hospital for their hip fracture; we attempted to find all patients at their first presentation; however, this was not always the case. Year of birth isn’t always known in The Gambia; therefore, there is likely to be some inaccuracy in calculated ages. This may be over- or under-estimation. The exact age- and sex-stratified population numbers for the study area were not released in the 2024 Gambian census, only for the total population; our estimates based on census proportions was a reasonable solution. The census did not contain the specific population for West Kiang; therefore, the slightly larger Mansakonko area population was used, possibly underestimating incidence. As Mansakonko contains both urban and rural populations and the population denominator is not precise for this region, an urban/rural comparison was not possible. We have assumed the hip fracture incidence rates in our study area are generalizable across the whole of The Gambia; data collected from both urban and rural regions increases the validity of this approach. Additionally, the hip fracture projections assume only the changing age and sex structure of the population will affect hip fracture incidence rates. Other factors, such as healthcare access, osteoporosis management, and lifestyle, such as dietary patterns and exercise, will also change over time and may influence rates.

In conclusion, this is the first study to prospectively characterize hip fracture incidence rates in The Gambia. Fragility fractures of the hip were common, indicative of bone fragility associated with osteoporosis. These findings support revision of the essential medicines list to include anti-osteoporosis medication, so that they can be appropriately deployed to reduce future fracture risk. Furthermore, solutions to improve pathways to care, including collaboration between TBS and allopathic healthcare should be sought. The number of hip fractures occurring across The Gambia is expected to nearly quadruple by 2054, which will place substantial burdens on the healthcare system. Calculation of hip fracture incidence will allow for the calibration of a Gambian specific FRAX tool^33^ that can be used in clinical settings throughout The Gambia, and if other countries wish, enable creation of proxytools for other West African countries. National planning and orthopedic services can utilize this study’s findings to find implementable solutions for better preparedness for the forthcoming rise in fragility fractures in older Gambian adults.

## Supplementary Material

Supplementary material is available at *Journal of Bone and Mineral Research* online.

Supplementary Figure 2

## Figures and Tables

**Table 1:** Characteristics of patients presenting to hospital with a hip fracture living in the study areas (Banjul, Kanifing, Brikama, and West Kiang), The Gambia, July 2022-July 2024.

	Number (total *N* =226)	%
**Age, mean (SD)**	71.2 (12.5)	
**Female sex**	146	64.6
**Reported place of residence^a^**		
**Lower River Region (LRR)**	7	3.1
**West Coast Region (WCR)**	137	60.6
**Banjul City Council (BCC)**	4	1.8
**Kanifing Municipal Council (KMC)**	78	34.5
**Presentation facility**		
**Private**	39	17.3
**Public**	145	64.2
**Traditional bone setter**	42	18.6
**Type of hip fracture**		
**Intracapsular**	57	25.2
**Pertrochanteric**	113	50.0
**Subtrochanteric**	21	9.3
**X-ray unavailable**	35	15.5
**Low impact mechanism of injury (ie, a fragility fracture)**	205	90.7
**Presentation delayed by > 2 wk from injury^b^**	68	30.1

a Reported place of residence aligns with the following local government authorities from the Gambian 2024 Housing and Population Census.

b *N* missing = 8. Abbreviations: LRR, Mansakonko; WCR, Brikama; BCC, Banjul; KMC, Kanifing.

**Table 2:** Hip fracture incidence in 5-yr age bands, overall and stratified by sex, for the study areas (Banjul, Kanifing, Brikama, and West Kiang) and The Gambia, 2024.

Five-year age bands (years)	Number hip fractures^a^ per year *(n)*	Sample area population^b ^(*n*)	Incidence rate per 100 000 person years	Relative risk ratio (95% CI)	Gambia total population^c^ (*n*)	Incidence rates per 100 000, age- standardized to Gambia population 2024^d^	Estimated number of hip fractures across The Gambia 2024
**Total population**							
**40-44**	3.0	76915	3.9		113 103		4.4
**45-49**	4.5	53 565	8.4	2.2 (0.51-9.48)	78 766		6.6
**50-54**	4.0	44 657	9.0	2.3 (0.51-10.28)	65 668		5.9
**55-59**	5.0	31 382	15.9	4.1 (0.98-17.16)	46147		7.3
**60-64**	14.0	27 283	51.3	13.2 (3.79-45.93)	40119		20.6
**65-69**	11.5	17 238	66.7	17.1 (4.8-60.92)	25 348		16.9
**70-74**	24.5	14 555	168.3	43.2 (13.03-143.25)	21 403		36.0
**75-79**	15.0	7306	205.3	52.6 (15.23-181.65)	10 743		22.1
**80-84**	16.5	5249	314.3	80.6 (23.56-275.71)	7719		24.3
**85**+	15.0	5332	281.3	72.1 (20.88-248.98)	7841		22.1
**Total over 40**	113.0	283 482	39.9	10.2 (3.24-32.1)	416 857	39.9	166.2
**Total over 50**	105.5	153 002	69.0	17.7 (5.62-55.76)	224 988	68.9	155.1
**Total over 65**	82.5	49 680	166.1	42.6 (13.46-134.8)	73 054	166.0	121.3
**Total males**							
**40-44**	2.0	37 517	5.3		55 168		2.9
**45-49**	4.0	27 443	14.6	2.7 (0.49-14.74)	40 355		5.9
**50-54**	2.5	23 049	10.8	2 (0.31-12.84)	33 893		3.7
**55-59**	2.0	16 337	12.2	2.3 (0.32-16.33)	24 023		2.9
**60-64**	5.5	13 749	40.0	7.5 (1.49-37.83)	20218		8.1
**65-69**	5.5	9136	60.2	11.3 (2.24-57)	13 434		8.1
**70-74**	6.5	7060	92.1	17.3 (3.55-84.38)	10 381		9.6
**75-79**	7.0	3471	201.7	37.8 (7.86-181.89)	5104		10.3
**80-84**	2.0	2172	92.1	17.3 (2.44-122.76)	3194		2.9
**85**+	3.0	2264	132.5	24.9 (4.16-148.95)	3329		4.4
**Total over 40**	40.0	142 198	28.1	5.3 (1.28-21.93)	209 099	28.1	58.8
**Total over 50**	34.0	77 238	44.0	8.3 (1.99-34.55)	113 576	44.0	50.0
**Total over 65**	24.0	24 103	99.6	18.7 (4.42-79.12)	35 442	99.5	35.3
**Total females**							
**40-44**	1.0	39 399	2.5		57935		1.4
**45-49**	0.5	26 121	1.9	0.8 (0.03-23.85)	38411		0.7
**50-54**	1.5	21 609	6.9	2.7 (0.22-33.9)	31 775		2.2
**55-59**	3.0	15 045	19.9	7.9 (0.82-75.94)	22 124		4.4
**60-64**	8.5	13 534	62.8	24.7 (3.11-196.13)	19 901		12.5
**65-69**	6.0	8102	74.1	29.2 (3.52-242.52)	11914		8.8
**70-74**	18.0	7495	240.2	94.6 (12.63-708.55)	11022		26.5
**75-79**	8.0	3835	208.6	82.2 (10.28-657.07)	5639		11.8
**80-84**	14.5	3077	471.2	185.7 (24.48-1408.47)	4525		21.3
**85**+	12.0	3068	391.1	154.1 (20.04-1184.77)	4512		17.6
**Total over 40**	73.0	141285	51.7	20.4 (2.84-146.77)	207 758	51.7	107.3
**Total over 50**	71.5	75 765	94.4	37.2 (5.17-267.72)	111412	94.4	105.1
**Total over 65**	58.5	25 577	228.7	90.1 (12.48-650.37)	37 612	228.8	86.0

a Mean annual number of hip fractures collected between July 2022 and July 2024.

b Population of Banjul, Kanifing, Brikama, and Mansakonko (inclusive of West Kiang) in 2024.

c Population of The Gambia in 2024.

d Annual incidence rate age-standardized to total Gambian population over 40 yr.

## Data Availability

Data are available upon reasonable request. Researchers can access participant level data via data.bris, provided ethical approvals are in place.

## Lay Summary

Little is known about how many fragility fractures occur in Africa. In West Africa, many are first managed by traditional bone setters (TBS), a role passed down through generations. We studied patients ≥40 yr with hip fracture presenting to hospitals or TBS over 2 yr in The Gambia. Hip fractures were more common in men than women 40-50 yr, due to high-energy trauma, and more common in women than men ≥60 yr, likely due to osteoporosis. Rates in The Gambia were similar to those for Black African adults elsewhere in Africa. Hip fracture cases are predicted to nearly quadruple in The Gambia by 2054.
