# Supplementary figures and images for: Age- and sex-specific incidence rates and future projections for hip fractures in The Gambia, West Africa, and comparison across four countries in Africa
Source: J Bone Miner Res. 2025 Sep 16;41(8):815–22. doi: 10.1093/jbmr/zjaf126 (PMC7619104; doi:10.1093/jbmr/zjaf126)

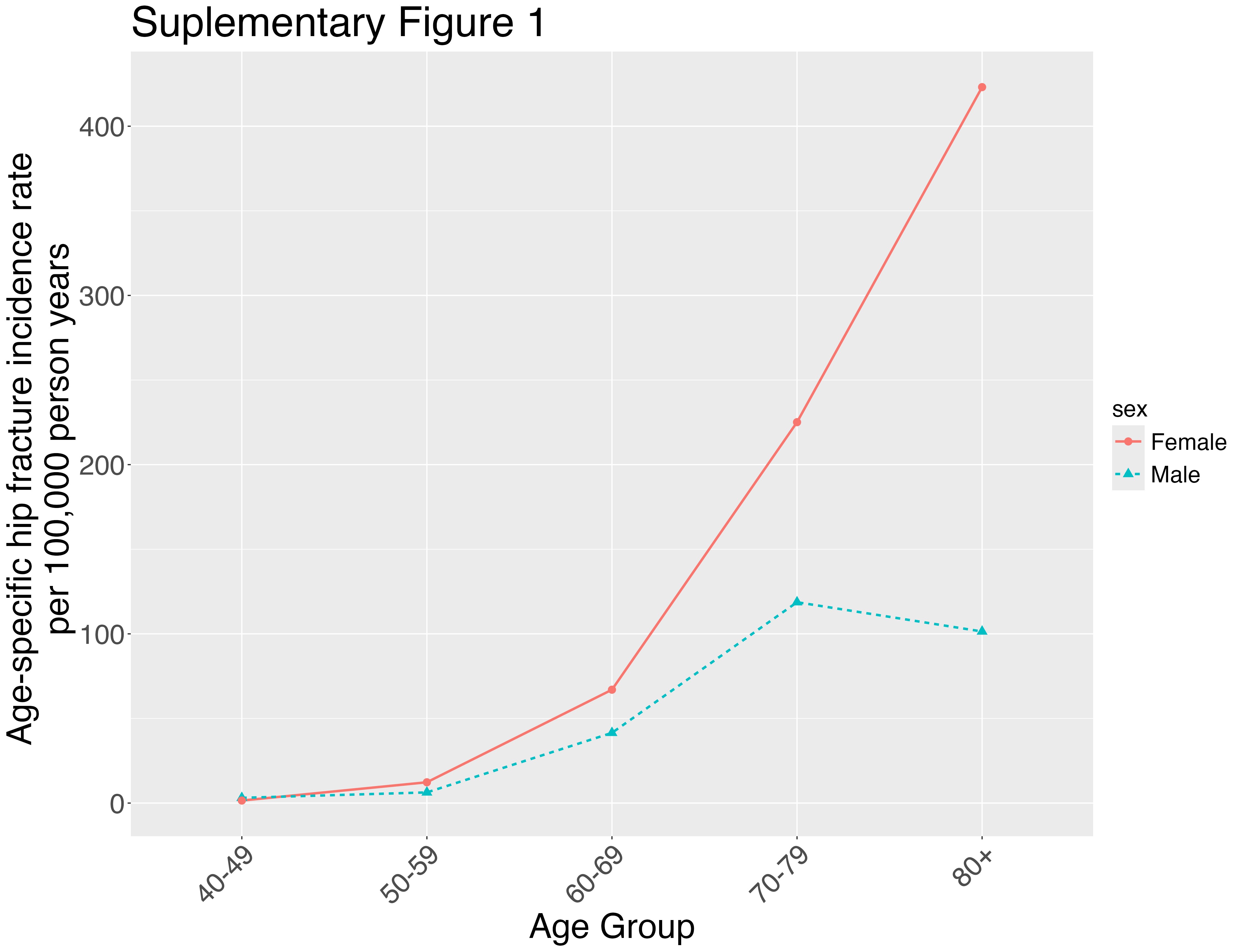

Supplement: Supp_Fig1_HF_incidence_Gambia_low_trauma_only_zjaf126 [file supp_fig1_hf_incidence_gambia_low_trauma_only_zjaf126.jpeg]

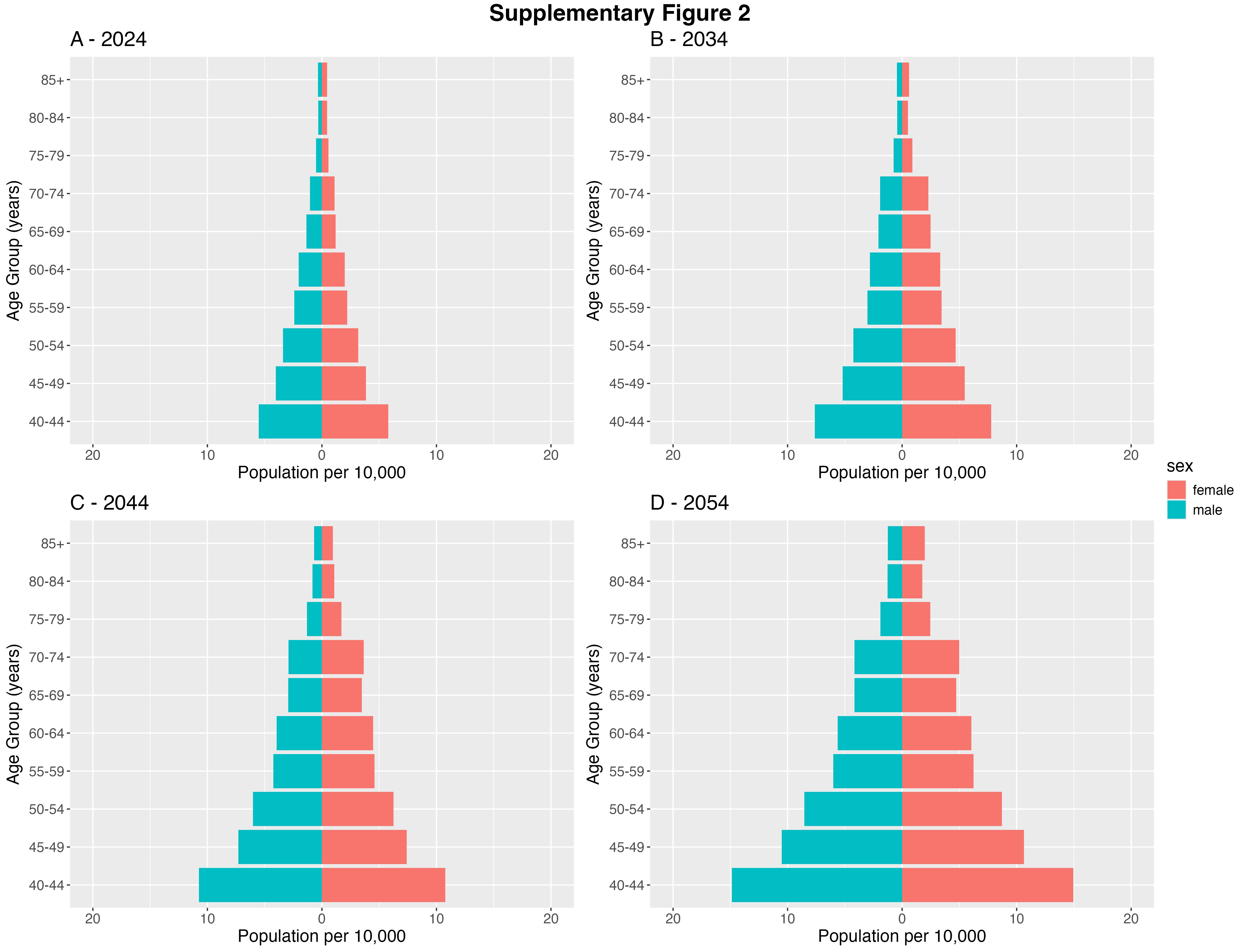

Supplement: Supp_Fig2_Population_pyramids_projections_zjaf126 [file supp_fig2_population_pyramids_projections_zjaf126.jpeg]
